# Spatially extensive microbial biogeography of the Indian Ocean provides insights into the unique community structure of a pristine coral atoll

**DOI:** 10.1038/srep15383

**Published:** 2015-10-20

**Authors:** Thomas C. Jeffries, Martin Ostrowski, Rohan B. Williams, Chao Xie, Rachelle M. Jensen, Joseph J. Grzymski, Svend Jacob Senstius, Michael Givskov, Ron Hoeke, Gayle K. Philip, Russell Y. Neches, Daniela I. Drautz-Moses, Caroline Chénard, Ian T. Paulsen, Federico M. Lauro

**Affiliations:** 1 Hawkesbury Institute for the Environment, Western Sydney University, Sydney, NSW, Australia; 2 Plant Functional Biology and Climate Change Cluster, University of Technology Sydney, Sydney, NSW, Australia; 3 Indigo V Expeditions, ONE°15 Marina, #01-01, 11 Cove Drive, Sentosa Cove, Singapore; 4 Department of Chemistry and Biomolecular Sciences, Macquarie University, Sydney, NSW, Australia; 5 Singapore Centre on Environmental Life Sciences Engineering (SCELSE), National University of Singapore, Singapore; 6 Division of Earth and Ecosystem Sciences, Desert Research Institute, Reno, NV, USA; 7 Department of Transport, Technical University of Denmark, Copenhagen, Denmark; 8 Costerton Biofilm Center, Department of International Health, Immunology, and Microbiology, Faculty of Health and Medical Sciences, University of Copenhagen, Copenhagen, Denmark; 9 Centre for Australian Climate and Weather Research, CSIRO, Aspendale, VIC, Australia; 10 VLSCI Life Sciences Computation Centre, University of Melbourne, Melbourne, VIC, Australia; 11 Genome Center, University of California, Davis, CA, USA; 12 Singapore Centre on Environmental Life Sciences Engineering (SCELSE), Nanyang Technological University, Singapore; 13 Asian School of the Environment, Nanyang Technological University, Singapore; 14 School of Biotechnology and Biomolecular Sciences, The University of New South Wales, Sydney, NSW, Australia

## Abstract

Microorganisms act both as drivers and indicators of perturbations in the marine environment. In an effort to establish baselines to predict the response of marine habitats to environmental change, here we report a broad survey of microbial diversity across the Indian Ocean, including the first microbial samples collected in the pristine lagoon of Salomon Islands, Chagos Archipelago. This was the first large-scale ecogenomic survey aboard a private yacht employing a ‘citizen oceanography’ approach and tools and protocols easily adapted to ocean going sailboats. Our data highlighted biogeographic patterns in microbial community composition across the Indian Ocean. Samples from within the Salomon Islands lagoon contained a community which was different even from adjacent samples despite constant water exchange, driven by the dominance of the photosynthetic cyanobacterium *Synechococcus.* In the lagoon, *Synechococcus* was also responsible for driving shifts in the metatranscriptional profiles. Enrichment of transcripts related to photosynthesis and nutrient cycling indicated bottom-up controls of community structure. However a five-fold increase in viral transcripts within the lagoon during the day, suggested a concomitant top-down control by bacteriophages. Indeed, genome recruitment against *Synechococcus* reference genomes suggested a role of viruses in providing the ecological filter for determining the β-diversity patterns in this system.

The Salomon Islands are located in the central Indian Ocean (~5° 19′ 0″ S, 72° 15′ 36″ E) approximately 320 nautical miles south of the equator (Fig. 1). They collectively delimit the borders of one of the atolls of the Chagos Archipelago and provide shelter to the few yachts crossing the Indian Ocean, which, if granted a permit, may anchor in the pristine waters of the lagoon. After the forced relocation of the native Chagossian people, Chagos Archipelago has remained uninhabited except for British and American military personnel located on Diego Garcia, more than 100 nautical miles south of Salomon. The Chagos Archipelago has now become the British Indian Ocean Territory (BIOT), which comprises seven atolls and over 1000 islands spread over approximately 640,000 square kilometers. BIOT was established as a marine protected area in 2010, banning extraction activity and doubling the size of the no-take zones worldwide^1^.

As a result of the limited anthropogenic input, its remote location and the proximity to the equator which shelters it from tropical cyclones, it has flourished as one of the healthiest coral reefs and has some of the cleanest waters in the world^1^,^2^.

While the Indian Ocean is generally considered oligotrophic, the enhanced productivity around the Chagos Archipelago^3^ is a result of the combined effects of Ekman pumping^3^,^4^ and ocean currents hitting the Chagos seamounts^3^,^5^.

While conservation efforts have been in place to maintain the integrity of the macro-faunal portion of the reef ecosystem, much less is known about the microbial community. Coral reefs, like most other ecosystems are intrinsically dependent on healthy microbial communities^6^,^7^.

Because of their abundance and functional role, microbes are the key providers of ecosystem services, acting as early sentinels of environmental change. Monitoring the marine microbiome is a useful parameter that can be used to detect changes and potential deleterious effects in any given ecosystem. Such information could be useful in efforts to better manage and protect fisheries, ecosystems and the greater ocean basin.

However, few studies have attempted to characterise the marine microbiome’s resilience and response to environmental perturbations, which as a first step, requires the establishment of baselines for diversity, functional capacity and activity of the autochthonous microbiome.

Here for the first time, the genetic diversity and transcriptional dynamics of the microbial community in the lagoon of the Salomon Islands is described. This dataset provides an insight into the unique microbiota of the lagoon, spanning viruses, bacteria and microbial eukaryotes and provides a baseline for future monitoring activities and conservation efforts. Moreover, this study was entirely conducted using a private sailboat serving as a proof of concept that ‘citizen oceanographers’ can contribute to our scientific perspective in a rigorous and meaningful way^8^.

## Results and Discussion

This large-scale microbial survey addressed the underexplored Indian Ocean, and encompassed the first microbial samples from the pristine Salomon Atoll. This was also the first large-scale ecogenomic study ever conducted aboard a private cruising yacht, with a small crew, using equipment and protocols that can be easily adapted for use aboard other sailboats^8^. Our study encompasses over 195 million RNA sequences and 17 million Small Subunit (SSU) ribosomal RNA tag sequences belonging to 5,264 unique Operational Taxonomic Units (OTUs), representing all three domains of life and relating to water samples spanning more than 39 degrees of latitude (Table 1). Data revealed the strong biogeographic partitioning present in this system and specifically identified the taxonomic drivers of shifts in microbial community composition across different oceanic regions and within the atoll.

### Biogeography of the Indian Ocean

**Figure 1:**
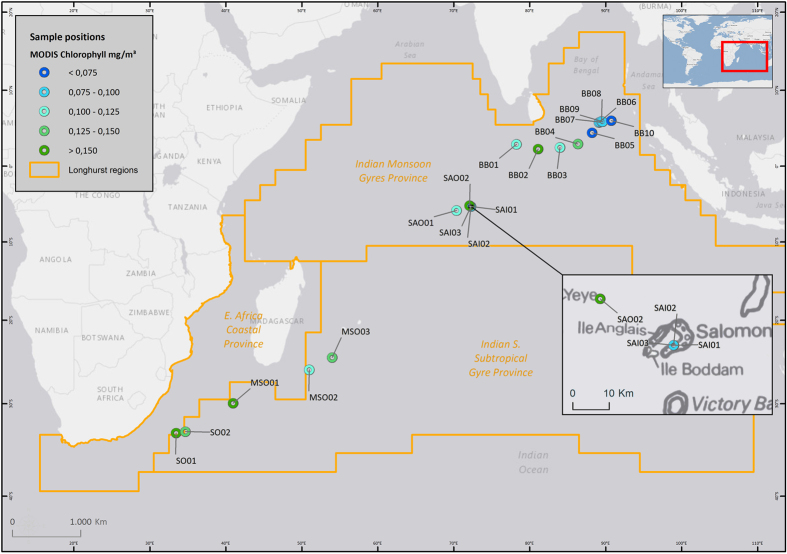
Location of the samples in relation to the Longhurst provinces^7^ of the Indian Ocean. The chlorophyll concentration of each sample was estimated from MODIS data using ESRI’s ArcGIS 10.3 Desktop as described in the methods.

**Figure 2:**
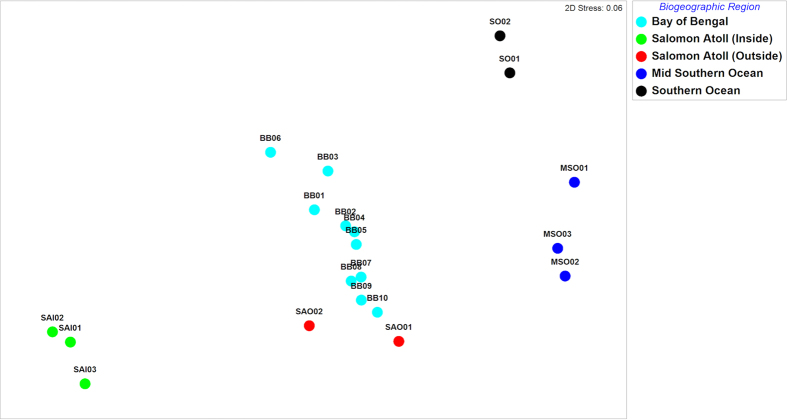
MultiDimensional Scaling (MDS) of Bray-Curtis similarity between OTU profiles from distinct water masses. Bay of Bengal* *= BB, Salomon Atoll (inside)* *= SAI, Salomon Atoll (outside)* *= SAO, Mid-Latitude Southern Ocean* *= MSO, Southern Ocean* *= SO. ANOSIM Global R* *= 0.93.

Microbial assemblages demonstrated strong biogeographic partitioning with taxonomic profiles reflecting water masses (Figs 1 and 2). Specifically, samples from the Southern Ocean (SO), mid-latitude Southern Ocean (MSO), Bay of Bengal (BB) and Salomon Atoll (SA) formed separate, highly discrete clusters. In all cases, clustering was statistically supported using ANOSIM (global R* *= 0.93, p < 0.05). These results indicate that microbial assemblage composition in the Indian Ocean is influenced by the physico-chemical composition and nutrient status of oceanic water masses and that the dispersal and relative abundance of taxa may be limited by hydrodynamic boundaries of currents^9^. The clustering of the samples is consistent with Longhurst’s marine biogeographic provinces^10^ defined using oceanographic data, measured chlorophyll, phytoplankton distributions and satellite data (Fig. 1, Table 1), further highlighting the tight coupling of microbial diversity with primary productivity and thermohaline properties. Few studies have investigated community-wide microbial biogeography in the context of Longhursts provinces, however, our results are consistent with the congruence of surface bacterioplankton communities within these regions in the eastern Atlantic Ocean^11^. This pattern highlights the usefulness of adopting common geographical frameworks across different trophic levels and datasets to elucidate global patterns in biogeography^9^.

Our observations of microbial provincialism are consistent with an increasing body of literature defining the discrete distributions of microbial communities in marine habitats^12^,^13^,^14^,^15^, individual marine microorganisms^16^,^17^ and taxa incorporated into ocean current models^18^,^19^.

The key bacterial and archeal taxa driving the dissimilarity between water masses were identified by SIMPER analysis (Supplementary Table 1) with differences between water masses consistently driven by shifts in SAR11 clades, *Synechococcus,* and *Prochlorococcus*. Previous studies have shown that, within these taxa, different phylotypes show a strong biogeographic distribution^9^. For example, samples from the BB were differentiated from the adjacent MSO due to increased abundances of *Synechococcus* and decreased *Prochlorococcus*. Overall this biogeographic pattern resulted in different ratios of autotrophic cyanobacteria in each water mass with increased proportion of *Synechococcus* relative to *Prochlorococcus* in the BB and *Prochlorococcus* being 6-fold more abundant than *Synechococcus* in the MSO. There were also shifts in SAR11 ecotype abundance between these two water masses with the SAR11 surface clade 1b being more abundant in the MSO and the surface clade 1a being more abundant in the BB. The high degree of dissimilarity between the BB and the Southern Ocean (SO) was driven by increased abundances of SAR86, *Altermonas* and *Synechococcus* in the BB cluster relative to the SO and an increase in SAR11 clade 1b in the SO. These phylotype abundance patterns related to temperature and latitude are in agreement with the study of Brown *et al.*^17^ who reported that the ratio of the surface clades of SAR11 1b over 1a is highest in waters with temperatures 19–24 °C^17^. SAR86 similarly has a reduced genome, well-adapted to oligotrophic conditions and shows temperature driven patterns in abundance in marine metagenomes^20^ with biogeographic patterns also reflecting substrate availability and niche competition with SAR11^20^. Marine *Synechococcus* populations display numerous phylogenetic clades and subclades^21^ representing ecotypes adapted to a variety of environmental conditions such as temperature and nutritional requirements and which vary in abundance in tropical coastal and open ocean water masses^9^. Similarly the global distribution of *Prochlorococcus* populations is determined by temperature, nutrient concentrations and photosynthetically active radiation (PAR), with this cyanobacterium tending to dominante in more oligtrophic conditions^9^ such as those found in the MSO.

Archaeal sequences were also top drivers of the biogeographic partitioning between water bodies (Supplementary Table 1) with Marine group II Archaea being more abundant in the BB samples relative to the MSO and higher in the MSO than the southernmost SO samples. Marine group I Archaea were top drivers of the dissimilarity between the SO and other water bodies due to their increased abundance in this cluster.

**Figure 3:**
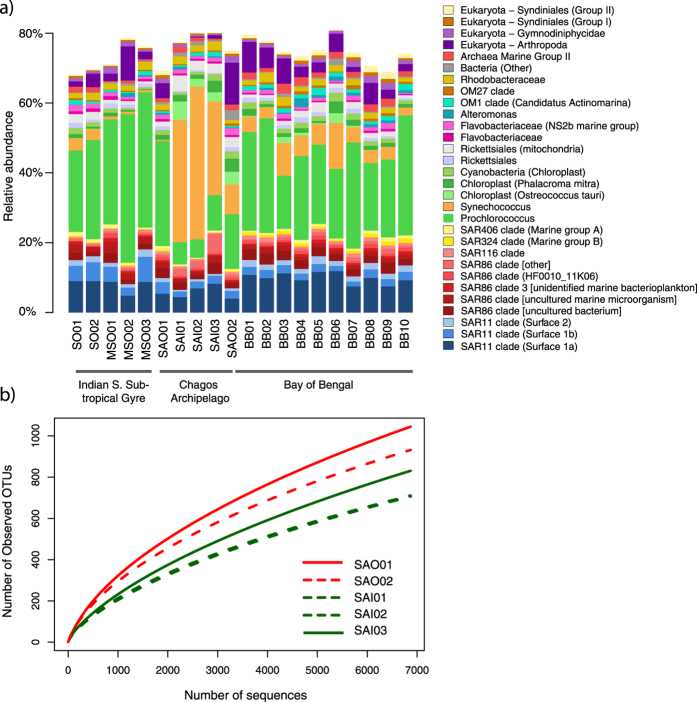
Structure and richness estimates of samples from the Chagos archipelago from Illumina 16S amplicons. (**a**) Relative abundance of most common taxa, (SILVA Database V. 119, Level 6; >0.5%). (**b**) Rarefaction curves of OTU observation inside and outside of Salomon Atoll. Subsampling was performed up to the level of rarefaction (6,871 sequences per sample).

The eukaryote sequences identified in the amplicon dataset ranged from 4% to 23% of the total number of amplicons at each site. Among the most abundant eukaryotic sequences were Arthopoda (Maxillopoda) and the endosymbiotic dinoflagellates *Syndiniales* (Fig. 3a). Chloroplast sequences belonging to the algae *Ostreococcus* and dinoflagellate *Phalacroma* were also abundant.

The abundant high-level groups (Supplementary Table 2) identified throughout the Indian Ocean include the Alveolates (Dinophyceae and Syndiniales); Metazoa (Maxillopoda); Stramenopiles (Bacilliarophyta and MAST); Hacrobia (*Chrysochromulina* and *Telonemia*) and Chlorophytes (*Ostreococcus*, *Micromonas* and *Bathycoccus*). Many of these eukaryotic taxa were major contributers to biogeographic patterns between water masses (Fig. 2, Supplementry Table 1). Specifically, *Bathycoccus* (chloroplast) was more abundant in the SO than in the BB and MSO groups and Arthropoda (Maxillopoda) was a major driver of the partitioning of BB samples away from other water masses, due to increased abundance in the northernmost parts of the transect (BB).

Overall, these specific biogeographic drivers, representing bacteria, archaea and eukarya, are abundant taxa in all samples (Fig. 3a) however they are also among the most dynamic in abundance, collectively driving the top 10% of dissimilarity. Despite this, the majority of the overall dissimilarity between water masses was driven by small contibutions from taxa across all relative abundances (Supplementary Table 1) indicating that a large proportion of the community responds to the environmental variablilty underpinning biogeographic patterns across this >4,000 km transect and suggesting shifts in ecological function related to these groups.

### Biogeographic partitioning of the Salomon Islands Atoll within Chagos Archipelago

Against the backdrop of mesoscale biogeographical patterns, the most striking demarcation is the unique structure of the microbial communities of the samples taken from within the lagoon of the Salomon Atoll. Ordination of abundance profiles (Fig. 2) demonstrated that samples from within the lagoon (SAI1-3) show highly discrete community composition when compared to surrounding Indian Ocean water masses. In particular, the samples did not cluster with samples located just adjacent to the lagoon (SAO1-2) despite constant water exchange and similar temperature and salinity (Table 1). These adjacent samples were more similar to Bay of Bengal samples (BB01-BB10) which clustered separately from the Southern Indian Ocean (SO01-SO02) and mid-latitude South Indian Ocean (MSO01-MSO03) samples. Similar patterns were observed using the weighted UniFrac metric (Supplementary Figure 1), with lagoon samples showing highly conserved phylogeny, which is unique to the atoll and distinct from surrounding waters. SIMPER analysis (Supplementary Table 1) indicated that the main drivers of the dissimilarity between the atoll and other samples was an increase in the cyanobacterial genus *Synechococcus* within the atoll, coupled to a reduction of *Prochlorococcus*, and shifts in the abundance of several oligotrophic SAR86 and SAR11 bacterial clades and eukaryotic sequences, in particular those belonging to the Arthropoda (Maxillopoda) (Fig. 3a). *Synechococcus* have previously been shown to play important roles in tropical lagoon systems and coral reefs playing a role in nitrogen cycling and forming the base of the food web via carbon fixation and grazing^22^.

Maxillopoda (crustacea and copepod) sequences are abundant in daylit surface waters from the Southern Ocean to the Bay of Bengal (Classified as Arthropoda at level 6 of Silva119). However, they are almost absent from the daytime samples in the lagoon but increase slightly at night. (Supplementary Table 2). The Dinophyceae are represented in all samples and clustered into at least 7 major OTUs including *Gymnodinium fusiforme,* which is preferentially found in ocean waters outside of the Salomon Islands lagoon. Syndiniales are the most diverse high-level group with 48 OTUs and 5 major groups, including Dino-Group-II-Clade-5 and Dino-Group-I clades that are present throughout the transect but show higher abundance in the low latitude samples.

The Chlorophytes *Micromonas* and *Ostreococcus* are detected within the lagoon during the day, with decreased abundance at night, and absent or present in low abundance in the ocean samples. *Chrysochromulina* (Hacrobia) is another phytoplankton species that shows higher abundance within the lagoon.

### Metatranscriptional profiling of the communities from Chagos Archipelago

**Figure 4:**
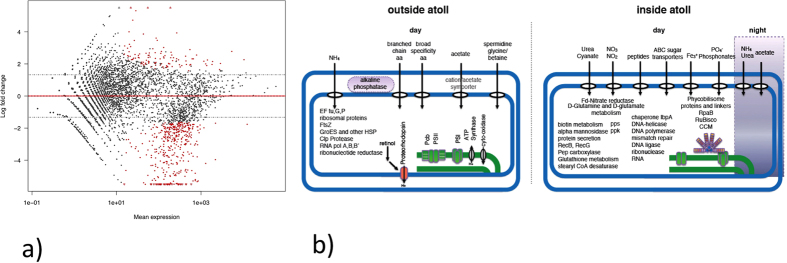
Metatranscriptional profiling of the community outside and inside the Salomon Atoll. (**a**) Scatter plot of log-fold change versus mean levels of transcript abundance between inside and outside water samples. The red points represents transcripts enriched or depleted in the community transcriptome under the statistical model used and the triangles are points that are outside the defined limits, in this case having a log fold value of +/− 5.5. The dashed lines indicate the log-fold cutoff of +/−1.6. (**b**) Cartoon depicting the major differences in transcript abundance between the outside (SAO1-2), inside day (SAI1-2) and inside night (SAI3) samples.

We performed a survey of community level gene expression in samples from within and outside the Salomon Islands lagoon (Fig. 4). Unsurprisingly, among the most actively transcribed gene categories were those related to photosynthesis (Photosystem I and II), peptidoglycan, protein and fatty acid biosynthesis. A differential abundance of phycobilisome antenna transcripts was observed and attributed to the higher abundance of *Synechococcus* within the lagoon. In contrast there was higher *pcbA* gene expression outside the lagoon, which can be attributed to the chlorophyll-binding light harvesting antenna of *Prochlorococcus*, which represented a much larger proportion of the phototrophic community outside of the lagoon (Fig. 3a). Similarly, a higher abundance of retinol metabolism genes attributed to SAR86 were enriched outside of the lagoon in concordance with a higher abundance of this clade^20^. Retinol is a precursor for assembling proteorhodopsin in photo-heterotrophic bacteria^23^ including SAR11 and SAR86.

Interestingly, the increased abundance in *Synechococcus* within the lagoon resulted in a corresponding enrichment in transcripts for exploitation of a wider range of nitrogen compounds. On average, more than 50% of the transcripts relating to nitrogen metabolism (K00284, K00366, K00367, K01455, K01501, K01673, K01725, K01915, K02575, K15576, K15578) could be taxonomically assigned to *Synechococcus* compared to less than 25% outside. Sequences of membrane transporters for urea, cyanate, nitrite/nitrate and peptides were enriched in the community transcriptome alongside genes in assimilation pathways for urea, cyanate, nitrate and glutamine, glutamate and glutathione metabolism. Outside the lagoon ammonia transport (*amt*) genes and broad specificity amino acid uptake ABC transporters comprised the majority of transcripts for nitrogen uptake genes. However, genes for spermidine and glycine-betaine transport systems were also more abundant in oceanic waters outside the lagoon. Spermidine has been shown to regulate the transcription of genes involved in carbon and nitrogen metabolism^24^ and spermidine transport genes have been found in high abundance in cultured marine bacterial genomes^25^. These observations suggest that spermidine may play an important role in regulating the nitrogen and carbon budgets of bacterioplankton. Spermidine binding proteins are also abundant in SAR11 metaproteomes from oligotrophic waters^26^. Glycine-betaine is additionally a nitrogenous osmoprotectant^27^ that has been suggested to play a role in nutrient cycling^28^. Overall these results suggest that the shift in community compostion results in a higher proportion of trascripts for nitrogen assimilation in the community inside the lagoon.

Genes involved in phosphorus uptake also showed differential patterns of transcript abundance with enrichment of organic phosphatases in oceanic waters adjacent to the lagoon and a potential phosphonate transport system enriched inside. Within the lagoon there is evidence of increased cycling of internal P into and out of polyphosphate stores with higher transcript abundance of polyphosphate kinase (*ppk*) and polyphosphatase genes. A recent study by Zhang *et al.*^29^ reported elevated concentrations of polyphosphate granules in the tissues of reef sponges. The polyphosphate was shown to come from cyanobacterial symbionts and suggested to have important implications in the recycling of phosphate in the reef ecosystem. The taxonomic assignment of the majority of the *ppk* transcripts to *Synechococcus* (91% inside the lagoon vs. 7% outside) suggests this might be a possible mechanism providing planktonic cyanobacteria in the lagoon ability to grow on polyphosphate as the sole source of P^30^.

The high proportion (up to 61%) of phototrophic sequences in transcript and SSU rRNA datasets suggests that conditions within the lagoon support enhanced primary productivity (Supplementary Figure 2). Recruitment of mRNA sequences to available genome sequences show the lagoon is inhabited by cyanobacterial populations closely related to *Synechococcus* sp. WH8109, a representative of marine Clade II(a) (average nucleotide identity +97% across 95.5% of the genome; Supplementary Figure 3). *Synechococcus* Clade II(a) is widespread across (sub)tropical oceanic waters^21^,^31^ but has been observed as a dominant phototroph in the nutrient rich waters of the Mauritanian upwelling^32^.

Against a backdrop of strong diel variation in *Synechococcus* transcript abundance, overall patterns in similarity between metatranscriptomes (Supplementary Figure 4) showed that the night sample from inside the lagoon was more similar to day samples from outside the lagoon. This suggested that the activity of *Synechococcus* during the day was a major driver of transcriptional differences between communities inside and outside the lagoon that was not observed at night, which is supported by the observation that genes involved in photosynthesis were among the main drivers determining this ordination (Supplementary Table 3). Interestingly, this pattern was not observed using RiboTagger abundance of transcribed SSU rRNA genes (Supplementary Figure 5), showing the same patterns as SSU rDNA OTU profiles, in which daytime and nighttime profiles were highly similar and clustered away from those outside of the lagoon. This indicated that the demarcation between night and day metatranscriptomes from the lagoon was not merely a reflection of the taxonomic composition of active microbes, but was a genome wide transcriptional response.

Samples within the lagoon were characterised by lower diversity than those adjacent (Fig. 3b), with a lower richness of OTUs occurring within the lagoon. This phenomenon might be analogous to the small island effect^33^ that suggests that species richness decreases with the decrease in habitat diversity below a certain threshold in island size. However, this would imply the existence of physical barriers to dispersal, which are unlikely due to the extensive mixing of water between the lagoon and the outside ocean. Analogous strong biogeographic patterns have been previously observed for Pacific coral atolls and attributed to ‘bottom-up’ forcing factors such as steep gradients in nutrient availability within lagoon waters^34^.

### Viral ecological filtering underlines the biogeographical singularity of the Salomon Islands.

**Figure 5:**
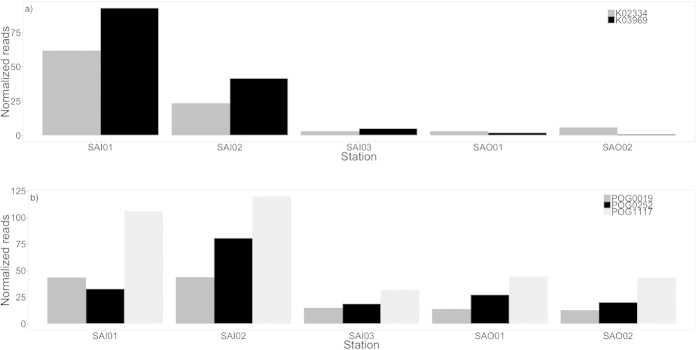
Transcript abundance of viral genes inside and outside the Salomon Island Atoll. (**a**) KEGG Orthology (KO): Grey bar represents the number of mRNA reads associated with DNA polymerase B bacteriophage-type (K02334) while the black bar represent the number of mRNA reads associated with phage shock protein A (K03969, *pspA*) for the station inside and outside the island atoll. The numbers of reads were normalised to 1 million reads using the total amount of reads. (**b**) Phage Orthologous groups (POGs): The dark grey bar represents the number of mRNA reads associated with T3/T7-like RNA polymerase (POG0019), the black bar represents the number of mRNA reads associated with terminase large sub-unit (POG0252) and the light grey bar represents the number of mRNA reads associated with a structural gene (POG1117) for the station inside and outside the island atoll. The numbers of reads were normalised to 1 million reads using the total amount of reads.

As an alternative or complementary top down explanation of reduced diversity and discrete biogeography within the lagoon, we report an increase in the expression of viral-related genes inside the lagoon (Fig. 5). Using the KEGG Orthology (KO) database, we observed within the lagoon more than 5-fold enrichment in transcripts associated with increased phage activity such as *pspA* (which encode phage shock protein A, K03969) and phage DNA polymerase (K02334) (Fig. 5a). In addition, a search of the mRNA against the database of Phage Orthologous Groups (POG)^35^ showed that transcripts encoding T3/T7-like RNA polymerase (POG0019), terminase large sub-unit (POG0252) and a structural protein (POG1117) were also significantly higher inside the lagoon (Fig. 5b; Supplementary Table 4). This suggests that phages might provide the ecological filter that drives the shift in cyanobacterial populations^36^,^37^.

Multiple lines of evidence support this hypothesis. First the observed increase in phage activity was only evident in the daytime samples, suggesting that it is linked to active primary production (Fig. 5), as cyanophage production is believed to also show a diel pattern^37^.

Second the identity of phage transcripts within the lagoon argues for the induction of prophages from within cyanobacterial genomes. Indeed, most of the hits for the phage DNA polymerase were similar to a phage SPO1 DNA polymerase–related protein that is found in sequenced *Synechococcus* genomes. It is likely that the steep transition from the oligotrophic open ocean to the highly productive environment of the lagoon would be hostile to pelagic populations of *Prochlorococcus* and *Synechococcus* and the exposure to higher amounts of UV light or the change in host abundance^38^ would trigger prophage induction. This is reflected in the more than 4-fold enrichment in POG1405 (high-light inducible protein) transcripts observed in the diurnal samples of the lagoon (Supplementary Table 4).

These observations underline that, unlike coastal waters^39^,^40^ where phage production is primarily due to lytic infection, rather than induction of lysogenic bacteria, the increase of viral-related genes expression might be the reponse of the induction of lysogenic pelagic cyanobacteria.

**Figure 6:**
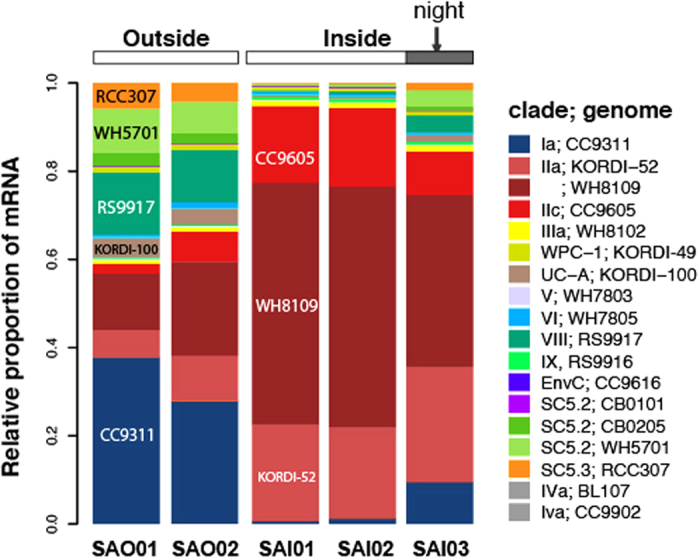
Relative abundance of *Synechococcus* genotypes inferred from genome recruitment of transcripts comparing inside and outside Salomon Islands.

Third, the lower diversity observed within the lagoon waters (Fig. 3b) supports the notion of periodic clonal sweeps^41^ with numerical dominance during the daytime by the most fit ecotypes, while at night the diversity increases (Fig. 3b) as new immigrant populations enter the lagoon through tidal and wind-driven mixing. In fact, the mRNA recruitments of the dominant *Synechococcus* genotypes showed pronounced shifts between day and night samples (Fig. 6) with immigrant populations, recruiting to clades I, VIII and sub clusters 5.2 and 5.3 genomes, being reduced during the daytime. The nocturnal immigration and diurnal selection of oceanic communities is also corroborated by amplicon data showing higher relative abundance in the night sample (SAI03) of heterotrophic taxa characteristic of waters from outside the lagoon (e.g. SAR11 and SAR86; Fig. 3a).

The precise nature of the forces maintaining the species–area relationships within the waters of the Salomon Islands is beyond the scope of this study, but given the role of diversity in maintaining the resilience of an ecosystem^42^, we posit that the microbial assemblages within the atoll are potentially more vulnerable to perturbations and anthropogenic impact than the surrounding oceanic ecosystem.

Strong patterns were observed in taxonomic composition across oceanic provinces and within the Salomon Islands. Gaining a deeper understanding of the function of these communities is essential to predict the ecological role of microorganisms in the system and measure their response to environmental change.

## Methods

### Sample collection

Samples were collected as part of the Indigo V Indian Ocean Expedition 2013^8^. A detailed description of sampling locations and basic water characteristics is reported in Table 1. Samples were collected from approx 2 m depth, into carboys that had been sanitised using household bleach (6% v/v sodium hypochlorite) and rinsed 4 times with local water. The water was then filtered through polyethersulfone Sterivex-GP 0.22 μm cartridges (catalog number SVGPL10RC, Merck Millipore) using an Athena portable peristaltic pump (Pegasus Pump Co., FL, USA) with 1.6 mL of RNAlater (Life Technologies, CA, USA) and stored at −80 °C until extraction. On average sample processing time took 45 minutes between the start of the pump collection and the addition of RNAlater.

The physical characteristics of the water column were recorded using an Open Hardware Arduino-based instrument with probes for temperature, conductivity (cell constant K = 1.0), pH. The instrument was calibrated using standard solutions (Atlas Scientific, NY, USA) and validated using a YSI sonde model 6600V2 (YSI Inc., OH, USA).

The following circuits were employed: Atlas Scientific EZO-pH, EDO-EC in combination with the Atlas Scientific ENV-TMP, ENV-40-pH, ENV-40-EC-1K.0 probes.

The hardware schematics and firmware can be downloaded with a BSD license at https://github.com/ryneches/Atlas

### MODIS Satellite data and generations of GIS plots

The AQUA/MODIS^43^ spatial and temporal spectroradiometry data for the Chlorophyll concentration was plotted with monthly temporal resolutions (http://neo.sci.gsfc.nasa.gov) corresponding to each station on the Indigo V transect. Temporal and spatial estimates data were used on days with cloud cover.

The extracted values were plotted with sample positions colored by chlorophyll concentration in mg/m^3^ on a grey canvas provided by ESRI (CA, USA) and overlaid by a map of the Longhurst provinces^10^,^44^ using ESRI’s ArcGIS 10.3 Desktop.

### DNA extraction and sequencing

DNA was extracted from the Sterivex filters using a modified Sucrose/SDS lysis method^45^. Briefly, Sterivex filters were thawed at room temperature, then the RNA later was pushed out of the filter and replaced with Phosphate Buffered Saline (PBS). Filters were washed twice with PBS. Sucrose Lysis Buffer^45^ and lysozyme were added and the filters were incubated at 37 °C with rocking for 30–45 minutes. 16 μl of RNase Cocktail (Life Technologies, CA, USA), 40 μl Proteinase K (20 mg/ml, Qiagen, CA, USA) and 40 μl 10% SDS were added to the filter and incubated at 55 °C for 1–2 hours. At that point the lysate was extracted with a standard Phenol-Chloroform-Isoamyl alcohol extraction and precipitated with 100% ethanol. Pelleted DNA was washed with ethanol (70% v/v) and resuspended in nuclease free water.

Amplicon libraries were generated by following Illumina’s 16S Metagenomic Sequencing Library Preparation Protocol, using 12.5 ng of template DNA per reaction. PCR cycles for the first PCR were reduced to 20 to avoid PCR biases from over-amplification. The following primers were used for the initial amplification, consisting of an Illumina-specific overhang sequence and a locus-specific sequence:

926wF_Illum: 5′- TCGTCGGCAGCGTCAGATGTGTATAAGAGACAGAAACTYAAAKGAATTGRCGG

1392R_Illum: 5′- GTCTCGTGGGCTCGGAGATGTGTATAAGAGACAGACGGGCGGTGTGTRC

This universal primer pair targets the V6-V8 hyper-variable regions of the 16S/18S Ribosomal RNA gene and has been shown to capture, in a single reaction, the microbial diversity of Archaea, Bacteria and Eukarya^18^.

PCR reactions were then purified with Agencourt AMpure XP beads (Beckman Coulter) and subjected to a second round of amplification for library barcoding according to Illumina’s recommendation.

Library quantitation was performed using Invitrogen’s Picogreen assay and the average library size was determined by running the libraries on a Bioanalyzer DNA 1000 chip (Agilent). Library concentration was normalised to 4 nM and the concentration was validated by qPCR on a ViiA-7 real-time thermocycler (Applied Biosystems), using qPCR primers recommended in Illumina’s qPCR protocol and Illumina’s PhiX control library as standard. The libraries were then pooled at equal volumes and sequenced in two lanes of an Illumina MiSeq V3 run at a final concentration of 4 pM and a read-length of 301 bp paired-end. The SSU amplicon data was deposited at NCBI under BioProject PRJNA281973.

### RNA extraction and sequencing

RNA was extracted using the Totally RNA kit (Life Technologies, CA, USA) with protocol modifications. After removing all residual RNAlater, the sterivex filter was washed with 25 mL of RNase free PBS, followed by the addition of 1.6 mL denaturation solution from totally RNA kit (Life Technologies, CA, USA). The filter was incubated at room temperature on a rotating platform for 30 minutes, inverted and incubated for a further 30 minutes. The denaturation solution was withdrawn, divided into two microfuge tubes and the remaining RNA extraction was completed as described in the Totally RNA manual (Life Technologies, CA, USA) following the phenol/chlorofom/isoamyl alcohol protocol. *In-vitro* ribosomal RNA depletion was not performed allowing for the analysis of the activity of different taxonomic groups using RiboTagger (see below).

Prior to library preparation, the quality of the RNA samples was determined by running the samples on a Bioanalyzer RNA 6000 Nano Chip (Agilent). Sample quantitation was performed using Invitrogen’s Ribogreen assay. To rule out DNA contamination, the RNA samples were also subjected to Invitrogen’s Picogreen assay.

Next-generation sequencing library preparation was performed by following Illumina’s TruSeq Stranded Total RNA protocol with the following modifications: the mRNA purification step was omitted and instead, 400 ng of total RNA were directly added to the elute-fragment-prime step. The PCR amplification step, which selectively enriches for library fragments that have adapters ligated on both ends was performed according to the manufacturer’s recommendation, but the number of amplification cycles was reduced to 12. Each library was uniquely tagged with one of Illumina’s TruSeq LT RNA barcodes to allow library pooling for sequencing.

Library quantitation was performed using Invitrogen’s Picogreen assay and the average library size was determined by running the libraries on a Bioanalyzer DNA 1000 chip (Agilent). Library concentration was normalised to 2 nM and the concentration was validated by qPCR on a ViiA-7 real-time thermocycler (Applied Biosystems), using qPCR primers recommended in Illumina’s qPCR protocol and Illumina’s PhiX control library as standard. The libraries were then pooled at equal volumes and sequenced in two lanes of an Illumina HiSeq2500 V1 rapid run at a final concentration of 10 pM and a read-length of 101 bp paired-end. RNA samples were sequenced on the Illumina HiSeq2500 with Rapid V1 chemistry. The raw metatranscriptomic datasets were deposited at NCBI under BioProject PRJNA281973.

### Analysis of microbial community composition and diversity

Illumina amplicons were analysed using the QIIME pipeline^46^ with standard protocols. Briefly, paired end sequences were joined using FastqJoin (http://code.google.com/p/ea-utils) and libraries were de-mutiplexed and quality filtered to truncate reads at positions with Phred scores <Q20 retaining only reads >75 bp and with <3 low quality bases and no N characters. Chimeras were detected and removed using USEARCH^47^ and *de novo* OTUs defined at 97% sequence similarity using UCLUST^47^. Taxonomy was assigned against the SILVA Database (v.119)^48^ using the Mothur taxonomy assigner^49^. Representative sequences from each OTU were aligned with the SILVA database using PyNAST^45^ and a phylogenetic tree constructed using FastTree 2^50^. This tree was used to measure the phylogenetic similarity between samples using weighted UniFrac^51^,^52^. The representative OTU sequences were also further analysed against the curated Protist Ribosomal Reference database^53^ and the taxonomy of eukaryotes was assigned using a UCLUST^47^ consensus assignment.

To ensure even sampling depth for subsequent analyses, OTU abundance data was rarefied to the lowest number of sequences for a sample (6871 sequences). Subsequent rarefactions at higher cut-offs did not significantly alter patterns in β-diversity or community composition (results not shown). Taxa abundance data derived from this table and the matrices of Bray-Curtis and UniFrac dissimilarity were visualised using PRIMER V6^54^ utilising Multidimensional Scaling (MDS) and SIMPER analysis^55^. α- and β-diversity metrics were calculated using QIIME.

### RiboTagger analysis of transcriptome data

Total RNA reads were analysed by RiboTagger (manuscript in preparation. Version 0.7.0. Available at https://github.com/xiechaos/ribotagger). Briefly, a position specific scoring matrix (PSSM) was used to scan all sequencing reads in order to detect sequences immediately next to the V4 region of 16S rRNA, and report a 33 nt tag sequence of the V4 region downstream of the PSSM matched position. Frequencies of each different V4 tags were normalised by the total sequencing reads covering a V4 tag. Multidimensional Scaling was then carried out on the V4 tag frequency data with Bray-Curtis dissimilarity using PRIMER V6^54^.

### In-silico rRNA depletion and transcriptome analysis

Total RNA reads were screened by dc-megablast against SILVA rRNA database (v. 115)^48^ to remove potential rRNA reads. The remaining reads were used to search against the NCBI non-redundant dataset using DIAMOND^56^. A Lowest Common Ancestor (LCA) algorithm, implemented in LCAmapper as part of mtools (http://ab.inf.uni-tuebingen.de/data/software/megan5/download/mtools.zip) was used to report functional composition of each dataset based on DIAMOND search results, referencing reads to KEGG Orthology (KO) identifiers^57^. Full results are reported in Supplementary Table 5.

We then generated read count matrices with orthologous genes index in rows and samples in columns, and further normalised these using the variance stabilising transformations implemented in DESeq^58^. We further aggregated orthologous gene data to pathways as defined by KEGG, and computed the average of per-KO normalised read count as an overall summary of pathway expression. Full results are reported in Supplementary Tables 6 and 7.

The *in-silico* rRNA-depleted metatranscriptomes were also searched against the POG database^35^ using RAPsearch2^59^. Statistically significant differences between the number of hits to each POG in a comparison of the inside day samples (SAI01-02) and outside samples (SAO01-02) were evaluated with the resampling approach of Rodriguez-Brito^60^ using 1,000 subsamples of 10,000 POG hits each at a significance threshold of 98%. Amongst the differentially abundant POGs, only those that had at least 1,000 counts across all samples and a viral quotient higher than 0.8 were considered for further analysis. The viral quotient (VQ) is a measure of the likelihood of finding a POG only in phage genomes^35^ and is calculated as the ratio:


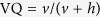


where

*v *= number of hits to viral genomes in NCBI/total number of viral genomes in NCBI

and

*h *= number of hits to prokaryotic genomes in NCBI/total number of prokaryotic genomes in NCBI (with hits to predicted prohage regions excluded).

Using viral quotient and minimum sample size cutoffs increased the confidence that the observed changes in POG hits were ‘true’ proxies for viral infection.

### Recruitment of RNAseq reads to representative genomes of *Synechococcus*.

The relative proportion of different *Synechococcus* genotypes within the mRNA reads was estimated with blastn^49^ against 18 available marine *Synechococcus* genomes using default parameters except for e-value of 1e^−5^ and a single best hit was reported for each read. Recruitment of mRNA reads against the WH8109 genome was performed with Bowtie2^61^ and the sam alignment was converted to a graph using BRIG^62^ and the coverage plots were produced in R.

## Additional Information

**How to cite this article**: Jeffries, T. C. *et al.* Spatially extensive microbial biogeography of the Indian Ocean provides insights into the unique community structure of a pristine coral atoll. *Sci. Rep.* **5**, 15383; doi: 10.1038/srep15383 (2015).

## Supplementary Material

Supplementary Figures and Table Legends

Supplementary Table 1

Supplementary Table 2

Supplementary Table 3

Supplementary Table 4

Supplementary Table 5

Supplementary Table 6

Supplementary Table 7

## Figures and Tables

**Table 1:** Sample location, collection date and environmental parameters.

ID	Latitude	Longitude	Date	Sunrise	Sunset	St	T	S	Chl	pH	WD
SO01	33°18.331′S	33°26.062′E	5/31/13	4:41	14:47	10:00	20.63	/	0.212	7.88	2591
SO02	33°09.163′S	34°40.656′E	5/31/13	4:36	14:42	21:30	19.96	/	0.125	7.54	1132
MSO01	29°56.725′S	40°56.317′E	6/5/13	4:06	14:24	9:45	21.29	/	0.212	7.73	4363
MSO02	26°02.308′S	50°58.611′E	6/9/13	3:19	13:52	9:00	22.12	/	0.184	7.83	/
MSO03	24°33.919′S	53°59.302′E	6/10/13	3:04	13:43	9:00	24.28	/	0.130	7.77	/
**SAO01**	5°51.686′S	70°21.761′E	9/14/13	1:12	13:16	7:30	28.15	34.12	0.117	/	3685
**SAI01**	5°20.605′S	72°15.578′E	9/16/13	1:04	13:08	8:00	29.39	34.94	0.079	7.73	23
**SAI02**	5°20.595′S	72°15.564′E	9/17/13	1:03	13:08	7:30	27.93	34.94	0.079	7.73	23
**SAI03**	5°20.595′S	72°15.564′E	9/17/13	1:03	13:08	18:00	27.1	34.56	0.079	7.56	23
**SAO02**	5°13.709′S	72°04.666′E	9/18/13	1:03	13:09	7:45	28.92	34.89	0.154	7.58	825
BB01	2°54.249′N	78°15.200′E	9/30/13	0:34	12:40	7:00	27.59	34.85	0.121	7.52	4404
BB02	2°16.476′N	81°06.718′E	10/1/13	0:23	12:28	6:00	27.76	/	0.204	/	4404
BB03	2°29.318′N	83°59.302′E	10/2/13	0:11	12:16	7:00	27.59	/	0.105	/	4499
BB04	2°56.229′N	86°21.500′E	10/3/13	0:01	12:06	6:00	27.76	/	0.149	/	4334
BB05	4°25.824′N	88°11.871′E	10/4/13	23:54 (–1)	11:58	6:00	27.93	/	0.067	/	4000
BB06	5°47.018′N	89°06.536′E	10/5/13	23:51 (–1)	11:53	3:00	/	/	0.085	/	3688
BB07	5°51.372′N	89°14.881′E	10/5/13	23:50 (–1)	11:53	6:00	27.9	/	0.091	/	3574
BB08	5°57.474′N	89°27.984′E	10/5/13	23:49 (–1)	11:52	10:00	/	/	0.08	/	3090
BB09	5°54.688′N	89°35.824′E	10/5/13	23:49 (–1)	11:51	12:00	/	/	0.08	/	3090
BB10	6°02.359′N	90°44.429′E	10/6/13	23:44 (–1)	11:46	4:00	/	/	0.071	/	2475

St = sample collection time; T = temperature (°C); S = salinity (PSS-78); Chl = chlorophyll (mg/m^3^); WD = water depth (m). Sunrise, sunset and sample collection time are given in coordinated universal time (UTC). RNAseq metatranscriptomes were generated for the samples highlighted in bold.
