# Supplementary material for: Spatially extensive microbial biogeography of the Indian Ocean provides insights into the unique community structure of a pristine coral atoll
Source: Sci Rep. 2015 Oct 20;5:15383. doi: 10.1038/srep15383 (PMC4611231; doi:10.1038/srep15383)
Supplement: Supplementary Figures and Table Legends [file srep15383-s1.doc]

**Supplementary information for Jeffries *et al.* “Spatially extensive microbial biogeography of the Indian Ocean provides insights into the unique community structure of a pristine coral atoll.”**

Thomas C Jeffries^1,2,3*^, Martin Ostrowski^3,4*^, Rohan B. Williams^5^, Chao Xie^5^, Rachelle M. Jensen^3^, Joseph J. Grzymski^3,6^, Svend Jacob Senstius^7^, Michael Givskov^8^, Ron Hoeke^9^, Gayle K. Philip^10^, Russell Y. Neches^11^, Daniela I. Drautz-Moses^12^, Caroline Chénard^12^, Ian T. Paulsen^3,4^, Federico M. Lauro^3, 12,13,14^.

*^1^ Hawkesbury Institute for the Environment, University of Western Sydney, Sydney, NSW, Australia*

*^2^Plant Functional Biology and Climate Change Cluster, University of Technology Sydney, Sydney, NSW, Australia*

*^3^ Indigo V Expeditions, ONE°15 Marina, #01-01, 11 Cove Drive, Sentosa Cove, Singapore*

*^4^ Department of Chemistry and Biomolecular Sciences, Macquarie University, Sydney, NSW, Australia*

*^5^ Singapore Centre on Environmental Life Sciences Engineering (SCELSE), National University of Singapore, Singapore*

*^6^ Division of Earth and Ecosystem Sciences, Desert Research Institute, Reno, NV, USA*

*^7^ Department of Transport, Technical University of Denmark, Copenhagen, Denmark*

*^8^ Costerton Biofilm Center, Department of International Health, Immunology, and Microbiology, Faculty of Health and Medical Sciences, University of Copenhagen, Copenhagen, Denmark*

*^9^ Centre for Australian Climate and Weather Research, CSIRO, Aspendale, VIC, Australia*

*^10^ VLSCI Life Sciences Computation Centre, University of Melbourne, Melbourne, VIC, Australia*

*^11^ Genome Center, University of California, Davis, CA, USA*

*^12^ Singapore Centre on Environmental Life Sciences Engineering (SCELSE), Nanyang Technological University, Singapore*

*^13^ Asian School of the Environment, Nanyang Technological University, Singapore*

*^14^ School of Biotechnology and Biomolecular Sciences, The University of New South Wales, Sydney, NSW, Australia*

**Supplementary Table 1 | Similarity Percentage (SIMPER) contribution of Level 6 taxonomy towards Bray-Curtis dissimilarity between distinct water masses.** Bay of Bengal = BB, Salomon Islands (inside) = SAI, Salomon Islands (outside) = SAO, Mid-Latitude Southern Ocean = MSO, Southern Ocean = SO.

**Supplementary Table 2 | Taxonomic classification and abundance of major Eukaryote groups (>10 OTUs).** Eukaryote OTUs were extracted from within the SSU rRNA representative sequences and then classified against the PR2 database. OTU abundances were derived from the rarefied OTU table that includes bacteria, archaea.

**Supplementary Table 3 | Similarity Percentage (SIMPER) contribution of KEGG orthologs towards Bray-Curtis dissimilarity between transcriptomic profiles.** Groupings refer to daytime samples within the Salomon Islands lagoon (high photosynthesis) vs daytime samples proximal to the lagoon and a night sample within (low photosynthesis). KO refers to KEGG Orthology (key provided as a tab).

**Supplementary Table 4 | mRNA sequence counts mapped to individual Phage Orthologous Groups (POG) and showing statistical differences between inside and outside Salomon Islands lagoon.** Details of the analysis are described in the methods section.

**Supplementary Table 5 | mRNA sequence counts mapped to individual KEGG orthology groups (KO) and statistical comparison of individual KO’s between samples inside and outside the Salomon Islands lagoon.**

**Supplementary Table 6 | Comparison of cumulative expression of KEGG pathways between daytime samples inside and outside Salomon Islands lagoon.**

**Supplementary Table 7 | Comparison of cumulative expression of KEGG pathways between daytime and nighttime samples inside Salomon Islands lagoon.**

**
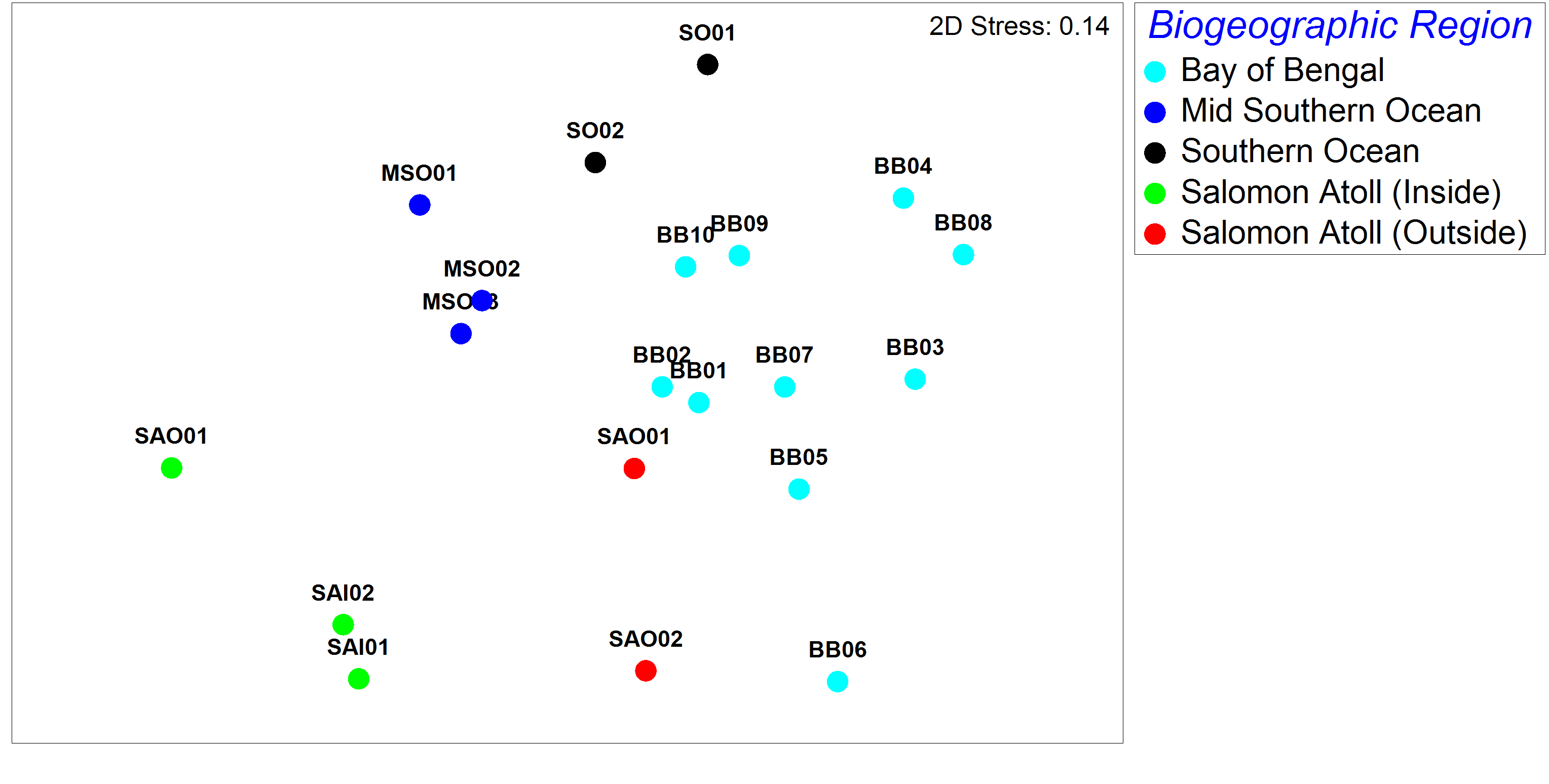
**

**Supplementary Figure 1 | MultiDimensional Scaling (MDS) of Weighted UniFrac similarity^43^ between OTU profiles from distinct water masses.**  Bay of Bengal = BB, Salomon Atoll (inside) = SAI, Salomon Atoll (outside) = SAO, Mid-Latitude Southern Ocean = MSO, Southern Ocean = SO.


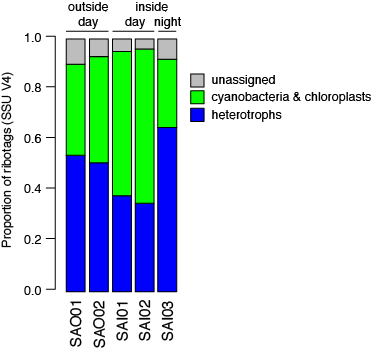


**Supplementary Figure 2 | Proportion of phototrophic versus heterotrophic V4 tags in RNAseq data as inferred by RiboTagger.**

Cyanobacteria and chloroplasts (green), heterotrophs (blue), unassigned (grey).


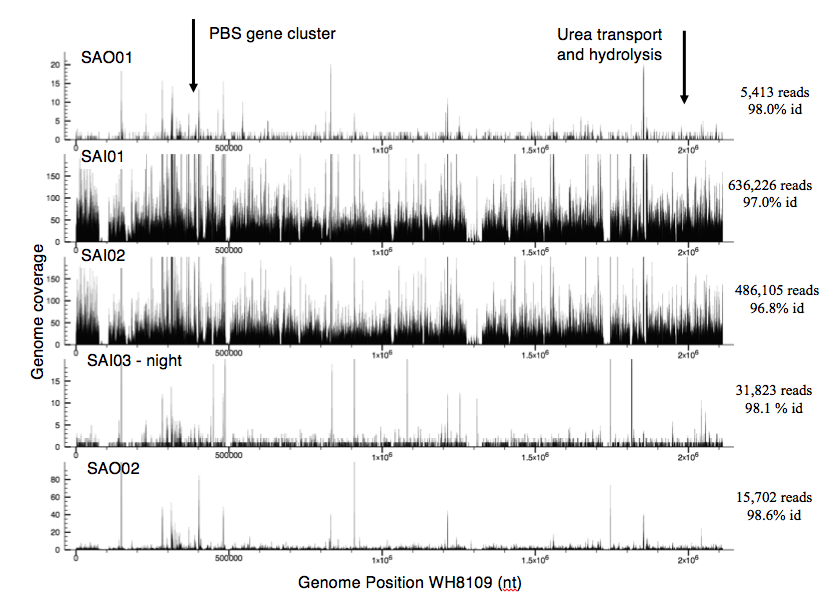


**Supplementary Figure 3 | Recruitment of RNAseq data to the genomes *Synechococcus* strain WH8109 comparing inside and outside Salomon Islands.** NB different scales on Y axes.

**Supplementary Figure 4 | Scatter plot comparisons between the RNAseq data mapped to individual KEGG orthology groups (KO) of different samples.** Salomon Atoll (inside) = SAI, Salomon Atoll (outside) = SAO


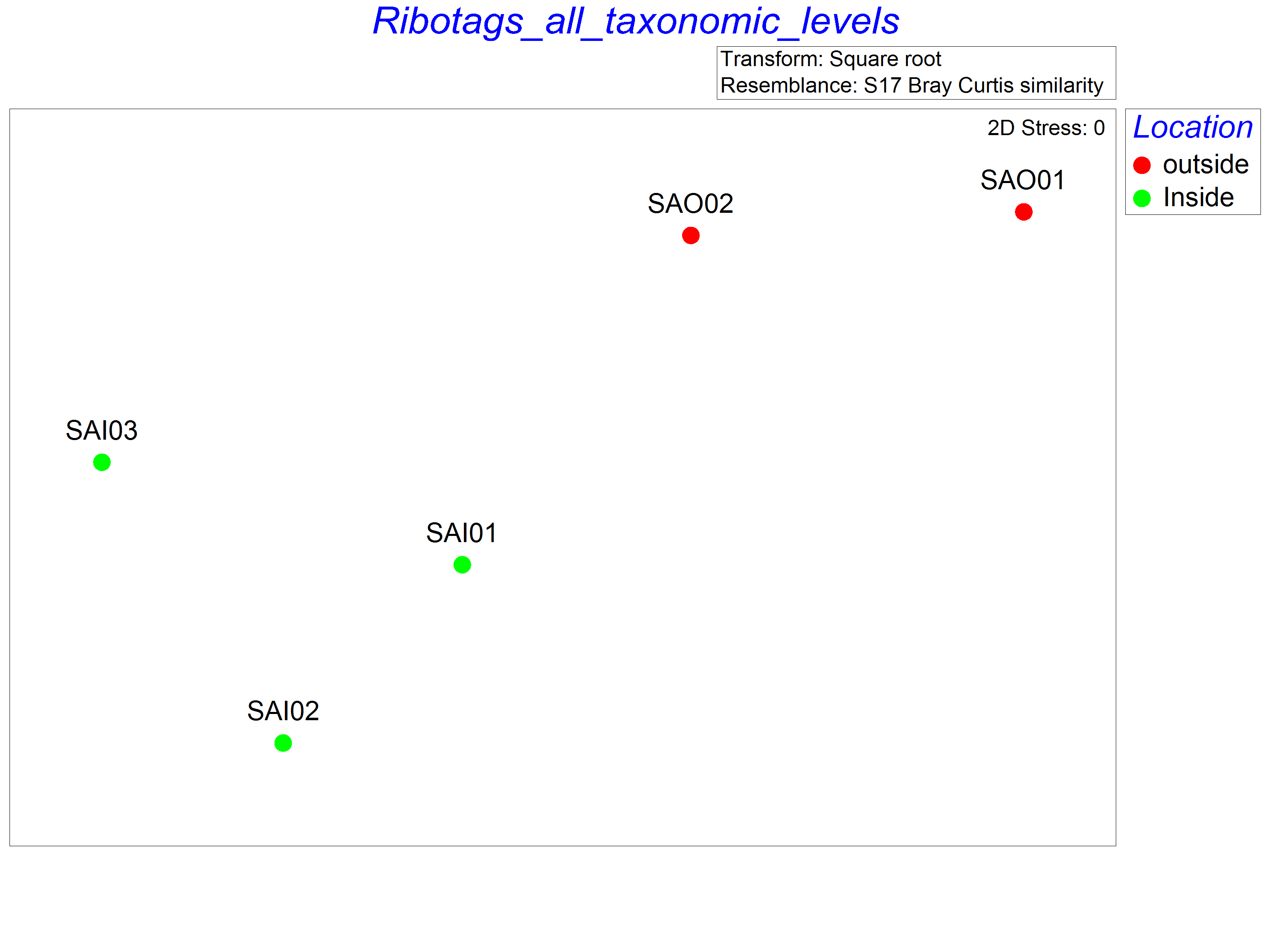


**Supplementary Figure 5 | MultiDimensional Scaling (MDS) of Bray-Curtis similarity between RiboTagger ribosomal V4 tag abundance profiles.** Tags were extracted from metatranscriptomic reads as described in materials and methods. Salomon Atoll (inside) = SAI, Salomon Atoll (outside) = SAO.
